# Exploring the association of household location and sociodemographic profile on dietary diversity in occupied Palestine: a serial cross-sectional study

**DOI:** 10.1186/s12889-025-23962-z

**Published:** 2025-08-08

**Authors:** Chesa Cox, Weeam Hammoudeh, Tracy Kuo Lin

**Affiliations:** 1 Institute of Global Health Sciences, University of California, San Francisco, 550 16th Street, Mission Hall, Box 1224, San Francisco, CA 94158 USA; 2 Institute of Community and Public Health, Birzeit University, PO Box 14, Birzeit, West Bank, Palestine; 3 Institute for Health & Aging, Department of Social and Behavioral Sciences, School of Nursing, University of California, 490 Illinois St., 12th Floor, Box 0646, San Francisco, CA 94143 USA

**Keywords:** Food consumption score, Food insecurity, Occupied Palestine, Conflict-affected settings, Undernourishment, West bank, Gaza strip, Undernourishment, Household food diversity

## Abstract

**Background:**

The prevalence of undernourishment is significantly higher in conflict-affected low- and middle-income countries (LMIC), compared to LMICs not experiencing conflict. Evidence suggests that in these settings households may adopt coping strategies such as consuming less nutritious food and thereby reducing food diversity to mitigate the impact of food insecurity. The long-term trend of food diversity in a protracted conflict setting has not been explored in detail due to challenges in collecting systematic and representative data in conflict-affected and fragile settings.

**Methods:**

This study examined food diversity – measured using food consumption scores (FCS) – among Palestinians in the Gaza Strip and the West Bank, utilizing a serial cross-sectional design to analyze a systematically random sampled dataset that was collected by the Palestinian Central Bureau of Statistics – from 2014, 2016, 2018, and 2020. We analyzed the distribution of household location by survey year and used multivariate linear regression to evaluate factors associated with changes in food consumption score.

**Results:**

Mean household FCS climbed from 71·9 in 2014 to 93·6 in 2016 but slipped to 73·4 in 2018 and 71·2 in 2020, signifying an overall decline in dietary diversity. For the West Bank, household location to the barrier, head of household gender (female), living in a refugee camp, and households with middle- or lower-income levels were associated with a reduction in FCS. For the Gaza Strip, households that reported minor mobility restrictions and middle- or lower-income levels were associated with a reduction in FCS.

**Conclusions:**

The findings elucidate the long-term impact of conflict on household food diversity, highlight a significant and worsening issue of food insecurity amongst Palestinians residing in the occupied Palestinian territory, and underline urgent need to address this critical issue and further protect vulnerable populations in conflict-affected regions.

## Background

The prevalence of undernourishment in conflict-affected low- and middle-income countries is significantly higher, ranging from 1·4 to 4·4% more on average, compared to their counterparts within the same economic classification that are not experiencing conflict [1]. In challenging environments affected by conflict, households may resort to coping strategies to withstand the negative externalities of conflict and ensure that household members receive sufficient food for consumption for as long as possible. These strategies may include consuming less nutritious food or limiting dietary variety so to focus on maintaining adequate caloric in-take – ultimately reducing the household’s dietary diversity [2–5].

The sustained conflict and Israeli military occupation have severely hindered productivity, food production, and availability in both the West Bank and the Gaza Strip (hereafter Gaza) – regions in the occupied Palestinian territory (oPt) [6]. These challenges are largely attributed to restrictions on Palestinian mobility, destruction of infrastructure, and constraints imposed on water and food markets by Israeli regulations [7]. While studies have explored food insecurity in Gaza and among Palestinian refugees in Lebanon, data on the West Bank remains scarce [8, 9]. Research has highlighted the associations between food insecurity (using the Radimer/Cornell food security scale) and various sociodemographic factors in Gaza [8]. These studies do not sufficiently address the long-term trend and nuanced interactions of household location and sociodemographic factors across the oPt.

**Fig. 1:**
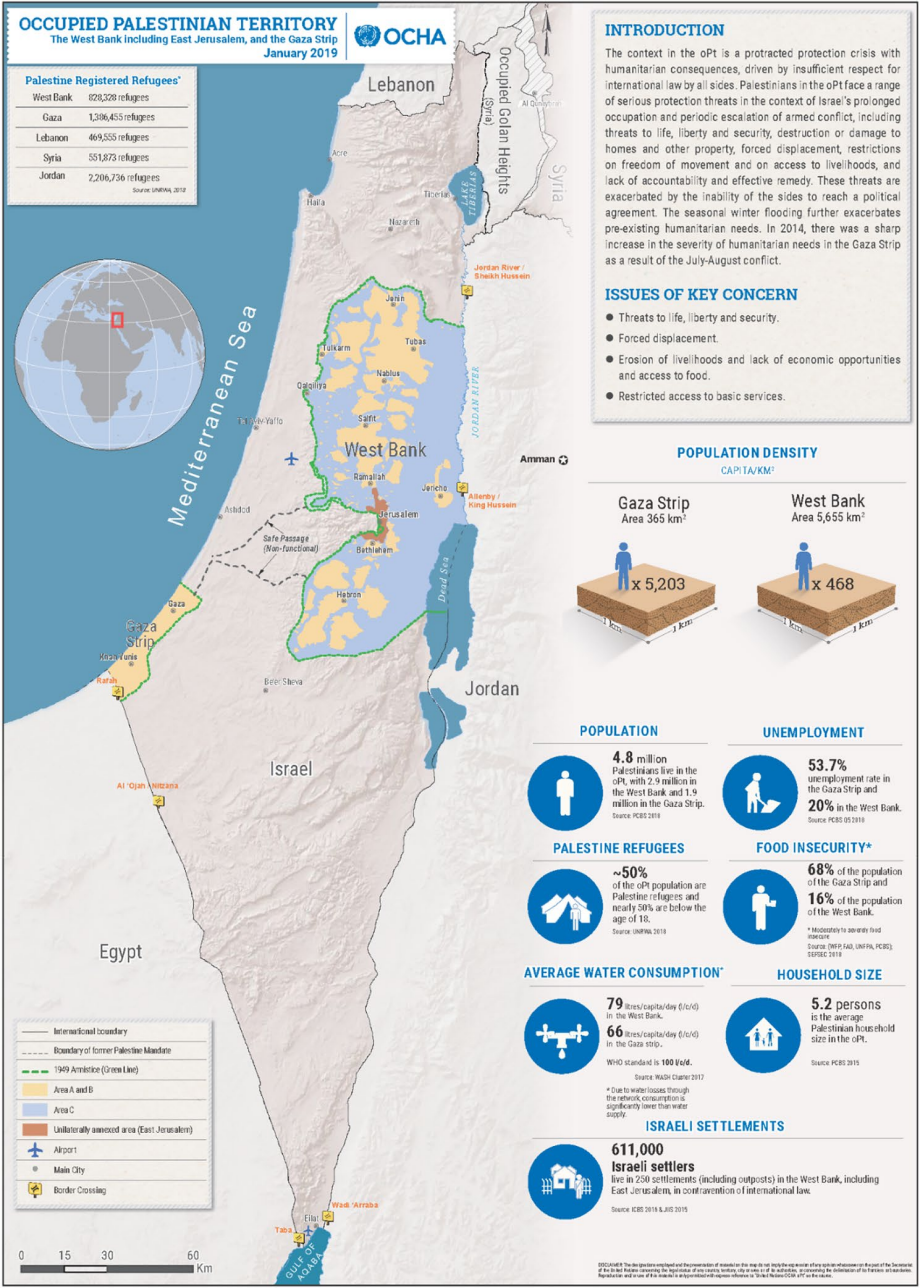
Occupied Palestinian Territory Map from 2019. Source: United Nations Office for the Coordination of Humanitarian Affairs (OCHA). Barriers and buffer zones are denoted by the green dotted line. The original figure is available at https://www.ochaopt.org/content/west-bank

The ongoing conflict in the oPt presents a unique opportunity to investigate factors associated with food insecurity. The protracted conflict and occupation of Palestine has fragmented communities in the region that is further complicated by military and administrative delineations such as Areas A, B, and C in the West Bank and buffer zones in Gaza (Fig. 1)

[10]. Areas A, B, and C in the West Bank are administrative divisions established under the Oslo Accords, reflecting differing levels of Palestinian and Israeli control. Area A is under full Palestinian civil and security control, encompassing major Palestinian cities which generally have better access to basic services than areas B and C. Area B is under Palestinian civil control and Israeli security control, primarily including rural areas and towns which have basic services, but quality and access is inconsistent. Area C, which constitutes the majority of the West Bank, is under full Israeli civil and security control, covering Israeli settlements, military zones, and much of the region’s agricultural land—facing the most significant challenges in accessing basic needs, such as food sources [11]. Buffer zones in Gaza are areas along the border with Israel where access is restricted or controlled. These zones are enforced by Israel and vary in size, with the most restrictive areas extending up to 300 m from the border, where entry is prohibited. Beyond this, access restrictions extend up to between 1,000 and 1,500 m, significantly limiting agricultural activity and construction. These divisions have created distinct geopolitical enclaves, each with varying levels of exposure to political and military strife.

Our study represents a unique serial cross-sectional analysis that builds on previous work and explores the trend of food consumption and association between household characteristics and dietary diversity in the context of a protracted conflict [7]. Previous studies found that living in areas with higher political and agricultural hardships—such as Area C or living outside the barrier in West Bank or living near buffer zones in Gaza—is associated with increased food insecurity and reduced dietary diversity [7, 12]. Furthermore, conflict reduced the overall resilience capacity of households to a certain extent, however, it induced aid response which may have mitigated food insecurity specifically in Gaza [12]. We add to previous work and hypothesize that the median food consumption score (FCS) trends downwards throughout the years, due to long-term exposure to conflict. The findings provide insight into the long-term impact of conflict and occupation on dietary diversity.

## Methods

### Study design

We conducted a serial cross-sectional study using data from 2014, 2016, 2018, and 2020 on the association of household sociodemographic factors on FCS in a sample of Palestinians living in the West Bank and Gaza, collected by the Palestinian Central Bureau of Statistics using the Socio-Economic Conditions Survey.

### Subjects and sampling

The target population for the survey included all Palestinian households with regular residency (households with residents that are registered with the Palestinian Civil Registry) in Palestine during the period of the surveys in 2014, 2016, 2018, and 2020. Head of households completed the questionnaire on socio-economic conditions and food consumption on behalf of the household. The survey was a representative sample of the Palestinian population, using a sampling framework that was based on enumeration areas, locality, and governorate levels. The sample design employed a three-stage stratified cluster systematic random sampling method where weights were calculated as the inverse of selection probabilities. Weights were assigned to enumeration areas based on their selection probability, then to households within these areas, and adjusted for attrition and appropriate household estimates by strata. This process corrected for selection biases and nonresponse, making the sample representative and ensuring the survey results are generalizable to the entire population. The Socio-Economic & Food Security Survey 2014: State of Palestine contains more information to the survey data, sampling strategy, and description [13]. As this is a serial cross-sectional study, the final dataset containing all 4 survey years may contain repeat households, as it is not possible to identify who had previously taken the survey.

### Measures

One of the commonly used measures for food diversity is FCS, which is an index score calculated using self-reported household consumption of eight food groups over seven days. The frequency of each consumed food group is then weighted by nutritional value and summed to produce a score that is classified into poor, borderline, or acceptable [14]. The FCS is used to monitor changes in food consumption and aid prioritization and formulate policies that address food security in conflict zones, which has been seen in Yemen, Syria and South Sudan [15–17].

Previous work demonstrated that location (i.e., living inside/outside the barriers in West Bank and near buffer zones in Gaza) and limited mobility are drivers of food insecurity, our variables of interest included region (West Bank vs. Gaza), locality type (urban, rural, refugee camp), proximity to barrier or buffer zones, self-reported household income, and extent that mobility has restricted the household. Because the head of household is typically the main decision maker and is responsible for financial support and welfare of the household, we have used the demographic variables of the head of household as a profile for the entire household. Demographic variables of interest such as head of household education, refugee status, gender, hours worked, and mobility were included in the analysis. Income amount and asset assessments were not consistent over survey years, making it difficult to quantitatively value household income, so household income used in this analysis was self-reported by head of household as very poor, poor, middle, or high income.

### Analysis

Food consumption score was calculated following the methodology provided by the World Food Programme (WFP) where households sum consumption frequencies by food group per week, multiply the sum from each group by a weight designated by WFP, and then added together to obtain a final FCS [18]. The maximum FCS a household can obtain is 112. Households with a score under 35 are considered as having an ‘unacceptable’ food consumption status. We used linear regression for both univariate and multivariate analysis to estimate significant mean differences in total Food Consumption Score (FCS) and p-value by sociodemographic characteristics at the 0.05 significance level. All models were estimated using the glm() function in R version [4·3.1] and p-values were derived from t-tests for individual coefficients. Final adjusted linear regression model was separated by region using data from 2020—West Bank and Gaza—to compare FCS amongst households with different proximity to barrier or buffer zones and to account for regional difference in exposure to conflict-related hardships.

## Resuts

### Study population

A total of 26,934 households responded to the surveys from 2014, 2016, 2018, and 2020. Households were excluded if they did not have any food consumption data to calculate the FCS, resulting in the analysis of data from 23,129 households (Table 1). It is important to note that due to the data collection process, we are unable to determine whether the same households participated in multiple years; some households may have been included in the dataset more than once. Overall, the median household size was 5 members. Heads of households were typically male (90·5%) and married (90·3%), where approximately half completed a secondary level education (46·5%), are not refugees (57·1%), and work over 35 h (50·5%). A higher proportion of households were in the West Bank (61·1%) and outside the barrier wall (56·8%), with 2020 having the highest proportion of households located in the West Bank (67·4%). The proportion of households within 1,000 m of the buffer zone in Gaza averaged 2·6% across all years. Majority of households were in urban settings (72·5%) and self-reported as middle class (70·9%). The median FCS across all households was an 80·0 (range: 3 to 112), showing fluctuations over the survey years with median scores of 81·0 in 2014, 84·0 in 2016, 79·0 in 2018, and 78·0 in 2020—all within the acceptable threshold.

**Table 1:** Descriptive characteristics of households (*N* = 23,129) amongst 4 waves (2014, 2016, 2018, 2020) of Socio-Economic conditions surveys done by the state of Palestine

	Overall	2014		2016		2018	2020	
	(*N* = 23129)	(*n* = 7900)		(*n* = 2195)		(*n* = 9925)	(*n* = 3109)	
Median FCS [Min, Max]	80 [3, 112]	81 [15, 112]		84 [16·5, 112]		79 [3, 112]	78 [10·5, 112]	
Household Locality								
Urban	16,764 (72·5%)	5606 (71·0%)		1424 (64·9%)		7491 (75·5%)	2243 (72·1%)	
Rural	3868 (16·7%)	1468 (18·6%)		451 (20·5%)		1438 (14·5%)	511 (16·4%)	
Refugee Camp	2497 (10·8%)	826 (10·5%)		320 (14·6%)		996 (10·0%)	355 (11·4%)	
Households in West Bank	14,138 (61·1%)	4892 (61·9%)		1255 (57·2%)		5897 (59·4%)	2094 (67·4%)	
Living in Area C	980 (4·2%)	283 (3·6%)		172 (7·8%)		420 (4·2%)	105 (3·4%)	
Inside barrier wall	993 (4·3%)	449 (5·7%)		121 (5·5%)		301 (3·0%)	122 (3·9%)	
Outside barrier wall	13,138 (56·8%)	4435 (56·1%)		1134 (51·7%)		5597 (56·4%)	1972 (63·4%)	
Households in Gaza	8991 (38·9%)	3008 (38·1%)		940 (42·8%)		4028 (40·6%)	1015 (32·6%)	
<1000 m from buffer zone	603 (2·6%)	269 (3·4%)		25 (1·1%)		272 (2·7%)	37 (1·2%)	
>1000 m from buffer zone	8385 (36·3%)	2737 (34·6%)		915 (41·7%)		3755 (37·8%)	978 (31·5%)	
Extent that mobility restrictions impacted household								
Not at all	13,270 (57·4%)	4005 (50·7%)		1595 (72·7%)		6185 (62·3%)	1485 (47·8%)	
Minor	5459 (23·6%)	1730 (21·9%)		354 (16·1%)		2403 (24·2%)	972 (31·3%)	
Very much	4173 (18·0%)	2100 (26·6%)		232 (10·6%)		1209 (12·2%)		632 (20·3%)
Don’t know	198 (0·9%)	59 (0·7%)		13 (0·6%)		126 (1·3%)	0 (0%)	
Head of Household Characteristics								
Median number of household members [Min, Max]	5·00 [1, 27]	6·00 [1, 25]		5·00 [1, 27]		5·00 [1, 27]	5·00 [1, 17]	
Male	20,931 (90·5%)	7148 (90·5%)		1990 (90·7%)		8966 (90·3%)	2827 (90·9%)	
Married	20,876 (90·3%)	7111 (90·0%)		1973 (89·9%)		8975 (90·4%)	2817 (90·6%)	
Highest level of education received: secondary	10,748 (46·5%)	3559 (45·1%)		1016 (46·3%)		4727 (47·6%)	1446 (46·5%)	
Works 35 + hours	11,689 (50·5%)	3694 (46·8%)		1220 (55·6%)		5247 (52·9%)	1528 (49·1%)	
Not a refugee	13,215 (57·1%)	4584 (58·0%)		1113 (50·7%)		5787 (58·3%)	1731 (55·7%)	
Received aid	14,439 (62·4%)	4519 (57·2%)		1334 (60·8%)		6660 (67·1%)	1926 (61·9%)	
Received food assistance	6510 (28·1%)	2427 (30·7%)		623 (28·4%)		2567 (25·9%)	893 (28·7%)	
*Household income								
Rich	902 (4·3%)	271 (3·4%)		··		461 (4·6%)	170 (5·5%)	
Middle	14,841 (70·9%)	5826 (73·7%)		··		6939 (69·9%)	2076 (66·8%)	
Poor	3743 (17·9%)	1368 (17·3%)			··	1765 (17·8%)	610 (19·6%)	
Very poor	1441 (6·9%)	434 (5·5%)			··	759 (7·7%)	248 (8·0%)	

*To calculate total proportion, did not include 2016 values in the denominator so *N* = 20,934

Table 2 reveals a consistent socio-economic and geographic gradient in household dietary diversity that persist across survey waves. Mean FCS peaked in 2016 (93.6 ± 26.3) but fell steadily thereafter, dipping below the 2014 baseline to 71.2 ± 24.5 in 2020, signaling a net 3-point decrease over six years despite remaining within the “acceptable” range overall. Households based in refugee camps, outside the West Bank barrier, and in Area C each scored ~ 2–3 points lower than urban or rural peers; in Gaza, proximity to the buffer zone showed no significant difference, underscoring location-specific vulnerabilities rather than a uniform conflict effect. A clear education and income gradient emerged: household heads with only elementary schooling averaged an FCS of 74.3, rising to 80.3 among those with post-secondary education, while FCS dropped from 81.9 in rich households to 68.3 in the very poor, with all trends significant at *p* < 0.001. Female-headed households, aid recipients, and the unemployed each averaged ~ 2–4 points lower than their counterparts, whereas part-time employment and rural residence were modestly protective. Finally, mobility constraints displayed a non-linear pattern: “minor” restrictions were associated with a significant 1.7-point deficit, yet the “very much” group showed a small, non-significant increase, hinting at possible residual confounding or limited power in this subgroup.

**Table 2:** Mean household FCS and standard deviation (SD) by location and sociodemographic factors. WFP designates scores less than the threshold of 35 as ‘unacceptable’ while the max score is 112

			Survey Year			
	Total	*P*-value	2014	2016	2018	2020
	(*N* = 23129)		(*n* = 7900)	(*n* = 2195)	(*n* = 9925)	(*n* = 3109)
Mean FCS (SD)	74·5 (21·9)		71·9 (13·7)	93·6 (26·3)	73·4 (23·1)	71·2 (24·5)
Location of Household						
Urban	77·1 (16·9)	ref	78·7 (14·3)	81·4 (14·7)	76·0 (18·0)	73·9 (19·6)
Rural	78·3 (15·4)	*<0·001	79·4 (13·1)	81·9 (12·8)	76·6 (17·3)	76·9 (17·1)
Refugee camps	75·4 (16·9)	*<0·001	75·6 (15·5)	79·0 (15·0)	75·9 (17·2)	70·4 (19·4)
Households in West Bank	77·4 (16·5)		79·9 (13·3)	79·3 (14·0)	76·1 (17·9)	74·3 (19·6)
Inside barrier wall	77·4 (16·0)	ref	81·8 (12·7)	77·7 (15·3)	69·7 (17·3)	80·3 (17·5)
Outside barrier wall	77·4 (16·6)	0·996	79·7 (13·3)	79·5 (13·9)	76·5 (17·9)	73·9 (19·7)
Area C (West Bank only)						
Yes	76·0 (17·0)	ref	78·6 (14·4)	80 (12)	74·2 (19·7)	69·9 (16·8)
No	77·8 (16·6)	*0·012	79·6 (13·4)	81·2 (14·6)	76·6 (17·7)	73·7 (19·9)
Households in Gaza	76·6 (16·9)		76·3 (15·5)	83·6 (14·5)	76·0 (17·7)	73·4 (18·3)
< 1000 m from buffer zone	75·4 (15·7)	0·183	73·1 (15·0)	77·8 (10·7)	77·5 (16·3)	75·9 (17·2)
* ≥* 1000 m and more	76·7 (17·0)	0·200	76·6 (15·5)	83·7 (14·5)	75·8 (17·8)	73·3 (18·4)
Extent that mobility restrictions impacted household						
Not at all	77·7 (16·8)	ref	79·9 (13·7)	81·3 (14·7)	76·2 (17·9)	74·1 (19·7)
Minor	76·0 (16·7)	*<0·001	77·1 (14·3)	80·1 (13·5)	75·8 (17·6)	72·8 (18·6)
Very much	77·0 (16·3)	*0·024	77·3 (14·9)	82·4 (13·2)	76·3 (17·3)	75·5 (19·2)
Head of Household Characteristics						
Gender						
Male	77·5 (16·6)	ref	78·9 (14·1)	81·6 (14)	76·5 (17·7)	74·3 (19·2)
Female	73·1 (17·4)	*<0·001	74·8 (15·7)	76·8 (16·7)	71·7 (18)	70·7 (19·5)
Marital Status						
Single/Divorced/ Separated/Widow	72·8 (17·6)	ref	74·9 (15·6)	76·9 (16·1)	71·0 (18·2)	70·0 (20·0)
Married	77·6 (16·5)	*<0·001	78·9 (14·1)	81·6 (14·1)	76·6 (17·7)	74·4 (19·1)
Highest level of education obtained						
Elementary	74·3 (17·1)	ref	76 (14·6)	78·2 (14·8)	72·9 (18·4)	70·6 (20·1)
Secondary	77·3 (16·6)	*<0·001	78·7 (14)	81·5 (14·1)	76·5 (17·7)	73·9 (19·3)
Intermediate and above	80·3 (15·7)	*<0·001	82·1 (13·6)	84·7 (13·4)	79·2 (16·6)	77·5 (17·7)
Employment						
Unemployed	76·2 (16·7)	ref	76·9 (14·4)	78·5 (15·8)	75·3 (18·4)	75·6 (17·9)
Working 1–24 h	78·9 (16·1)	*<0·001	81 (13·1)	82·9 (13·4)	77·7 (17·1)	74·6 (19·4)
Working 35 + hours	74·9 (17·2)	*<0·001	76·1 (15·1)	79 (14·9)	73·8 (18·3)	72·5 (19·4)
Refugee Status						
Not a refugee	76·7 (16·8)	ref	79·1 (13·7)	80·4 (14·2)	75·1 (18·1)	73·7 (19·5)
Refugee	77·6 (16·6)	*<0·001	77·8 (15)	81·9 (14·6)	77·4 (17·2)	74·3 (18·9)
***Received Aid***						
Yes	75·6 (16·8)	ref	75·8 (15·1)	81·1 (15)	75·3 (17·8)	72 (18·9)
No	78·0 (16·5)	*<0·001	80·5 (13·3)	81·2 (13·9)	76·4 (17·8)	75·2 (19·3)
***±Household income***						
Rich	81·9 (15·5)	ref	85·8 (10·2)	··	80·5 (16·6)	79·5 (17·9)
Middle	78·4 (15·8)	0·058	80·4 (13)	··	77·6 (16·9)	75·9 (18·4)
Poor	71·6 (17·9)	*< 0·001	72·4 (15·4)	··	72 (18·9)	68·7 (19·8)
Very poor	68·3 (19·6)	*<0·001	68 (17·7)	··	68·8 (20)	67·7 (21·3)

± Survey for 2016 did not include household income question, so overall mean *FCS* calculation uses data from 2014, 2018, and 2020

Linear regression analysis of the 2020 survey data revealed regional differences in the factors associated with changes in food consumption scores (FCS) across 3,109 households in the West Bank and Gaza. Among the 2,094 households in West Bank, the unadjusted model indicated that living outside the barrier was associated with a significant decrease of FCS by 6·37 points (*p* < 0·001; 95% CI: −9·95 to −2·79) (Table 3). The final adjusted model for West Bank includes location of household to barriers, locality, mobility, household income level, and head of household sex, education, and employment, and refugee status. The adjusted model also suggests that living outside the barrier is associated with a decrease in FCS by 5·51 points (*p* = 0·002; 95% CI: −9·10 to −2·08). Households living in refugee camps are also associated with a decrease in FCS of 7·04 points (*p* < 0·001; 95% CI: −10.12 to −4·00). Household income level had a negative linear relationship with FCS—where middle income households were associated with a decrease in FCS of 2·61 points (*p* = 0·026; 95% CI: −5·81 to 0·59), poor households with a decrease of 13·01 points (*p* < 0·001; 95% CI: −16·89 to −9·12) and very poor households with a decrease 19·61 points (*p* < 0·001; 95% CI: −26·67 to −12·47). Education level of household heads had a positive linear relationship with FCS, where secondary and intermediate and higher education levels were associated with increases in FCS of 2·45 (*p* = 0·016; 95% CI: 0·45 to 4·42) and 5·27 (*p* < 0·001; 95% CI: 2·97 to 7·54), respectively. Those with a refugee status was also associated with an improvement of 5·28 points in FCS (*p* < 0·001; 95% CI 3·21 to 7·37). Households headed by females were associated with a decrease of 2·51 points that was marginally not statistically significant, but improved model fit (*p* = 0·093, 95% CI: −5·42 to 0·42). The extent of mobility restrictions impacting households as a predictor improved model fit but was not statistically significant with “minor” mobility restriction having an associated decrease of 0·68 (*p* = 0·470; 95% CI: −2·56 to 1·18) and “very much” mobility restriction having an increase of 0·76 FCS points (*p* = 0·490; 95% CI: −1·39 to 2·92).

**Table 3:** Crude and adjusted linear regression results for selected variable by region (West bank and Gaza) with variables of interest associated with change in FCS using 2020 survey data (*n* = 3109). The unadjusted model for West bank is glm(totalFCS ~ barrier), while the adjusted is glm(totalFCS ~ barrier + mobility + head of household sex + education + employment + locality + household income + refugee status). The unadjusted model for Gaza is glm(totalFCS ~ mobility), while the adjusted is glm(totalFCS ~ mobility + edu + marital status + household income)

	Crude Coefficient	Crude *p*-value (95%CI)		Adjusted Coefficient	Adjusted *p*-value (95%CI)
West Bank (*n* = 2094)					
Intercept	86·65			74·7	
Living inside barrier	ref			ref	
Living outside barrier	−6·37	*<0·001 (−9·95 – −2·79)		−5·51	*0·002 (−9·10– −2·08)
Extent that mobility restrictions impacted household					
Not at all				ref	
Minor				−0·68	0·47 (−2·56–1·18)
Very much				0·76	0·49 (−1·39–2·92)
Head of household sex					
Male				ref	
Female				−2·51	0·093 (−5·42–0·42)
Head of household education					
Elementary				ref	
Secondary				2·45	*0·016 (0·45–4·42)
Intermediate and above				5·27	*<0·001 (2·97–7·54)
Locality					
Urban				ref	
Rural				2·90	*0·004 (0·95–4·81)
Refugee camps				−7·04	*<0·001 (−10·12 – −4·00)
Household income					
Rich				ref	
Middle				−2·61	*0·026 (−5·81–0·59)
Poor				−13·01	*<0·001 (−16·89 – −9·12)
Very Poor				−19·61	*<0·001 (−26·67– −12·47)
Refugee status					
Not a refugee				ref	
Refugee				5·28	*<0·001 (3·21–7·37)
Gaza (*n* = 1015)					
Intercept	73·90			77·59	
Extent that mobility restrictions impacted household					
Not at all	ref			ref	
Minor	−3·40	*0·010 (−5·97 – −0·83)		−3·50	*0·007 (68·98–86·19)
Very much	2·85	0·059 (−0·11–5·80)		2·92	0·050 (−0·00–5·84)
Head of household education					
Elementary				ref	
Secondary				2·00	0·170 (−0·86–4·86)
Intermediate and above				4·06	*0·014 (0·83–7·29)
Marital status					
Single/Divorced/Separated/ Widow				ref	
Married				4·0	0·049 (0·02–8·05)
Household Income					
Rich				ref	
Middle				−7·57	0·054 (−15·27–0·13)
Poor				−10·47	0·009 (−18·2 – −2·67)
Very Poor				−13·04	0·001 (−21·01 – −5·07)

~In 2020, the median of household *FCS* in West Bank is 79·5 (*SD* 19·6), median of household *FCS *in Gaza is 76·5 (*SD* 18·4). Linear regression model was separated by region using data from 2020—West Bank and Gaza—to compare *FCS* amongst households with different proximity to barrier or buffer zones and to account for regional difference in exposure to conflict-related hardships

In Gaza, the proximity to the buffer zone was not significantly associated with changes in FCS. However, in the unadjusted, univariate analysis, the extent that mobility restrictions impacted household was statistically significant—where “minor” impact is associated with a decrease in 3·40 FCS points (*p* = 0·010; 95% CI: −5·97 to −0·83). For Gaza, the final adjusted model includes impact of restrictions on household mobility, household income level, and head of household education and marital status. Intermediate education and above was significantly associated with higher FCS; with an average increase of 4·06 FCS points (*p* = 0·014; 95% CI: 0·83 to 7·29). The adjusted model also suggests that being married was associated with an increase in FCS by 4·0 points (*p* = 0·049; 95% CI: 0·02 to 8·05), compared to those who were single, divorced, separated, or widowed. Household income level was negatively associated with food consumption score with poor and very poor being significantly associated with decreases of 10·47 (*p* = 0·009; 95% CI −18·2 to −2·67) and 13·04 (*p* = 0·001; 95% CI −21·01 to −5·07) FCS points, respectively.

## Discussion

The findings of this serial cross-sectional study shed light on the complex interplay between household location, head of household demographics, and changes in FCS in the oPt. Over the years, the median FCS remained within the acceptable threshold but had a declining trend from 2016 to 2020 (Table 1). This pattern suggests a gradual decrease in dietary diversity, likely due to conflict from the Israeli occupation impacting the economy, agriculture, and overall access to food. The increase in the proportion of households reporting unacceptable or borderline FCS from 0·4% in 2016 to 4·0% in 2020 demonstrates an increase in food insecurity over a relatively short period of four years (Table 2). This trend warrants attention from policymakers and stakeholders to mitigate further declines in food access.

The data reveal regional differences in factors that are associated with changes in FCS using 2020 data. In the West Bank, location of household to barrier, restrictions on mobility, locality, household income, and head of household gender, education level, and refugee status are associated with changes in FCS. In Gaza, restrictions on mobility, household income, and head of household education and marital status are associated with FCS changes. This disparity can be attributed to several factors including geopolitical tensions, economic sanctions, and infrastructural challenges that are caused by conflict that uniquely affect each region [7].

In the West Bank, living outside the barrier, living in a refugee camp, minor mobility restrictions on household, household income, and households with female heads were associated with a decrease in FCS from a baseline value of 74·7. Households who had male heads were associated with an increase in FCS than their counterparts, which may reflect societal norms and structures that favor men in terms of employment opportunities and social stability [19, 20]. The correlation between higher educational attainment and improved FCS underscores the importance of education in improving dietary diversity and food security, possibly through greater knowledge of nutrition or greater job opportunities that improve access to food through improved household income. Households who identify as middle income, poor, and very poor had an increasing negative association with FCS in our multivariate analysis, indicating that interventions directed towards those of lower socioeconomic status may have a bigger impact in improving FCS. Household locality had an impact on food diversity; using urban households as the reference, rural households were associated with an increase in FCS while refugee camps were associated with a decrease in FCS. Rural households may have greater access to farms and agricultural resources, which allows household members to maintain their diet and FCS. Households in refugee camps have been recently displaced and have limited access to food supplies, which would decrease their household FCS. Contrasting with the association of FCS and refugee camps, household heads that self-reported as refugees were associated with a statistically significant increase in FCS. This may be due to refugees receiving food assistance from humanitarian programs, which could improve FCS; this finding is aligned with the evidence generated by Brück et al., suggesting the impact of aid in mitigating food insecurity [12].

In Gaza, minor mobility restrictions on households with middle, poor, and very poor income levels were associated with a decrease in FCS from a baseline value of 77·59. The minor mobility restrictions in Gaza have a bigger negative impact on changes on FCS compared to West Bank as indicated by the larger coefficient—suggesting that mobility in Gaza may be a bigger issue than in the West Bank. Like West Bank, there was an increasing negative association with FCS from middle to poor and very poor income levels, which suggests a similar intervention providing food assistance to households of lower socioeconomic status would target households vulnerable to food insecurity. Households where the head is married and/or have higher than a secondary education were associated with positive changes in FCS. Being married can improve FCS as the combined income, shared responsibilities, social support, and economies of scale within a married household often lead to more consistent and nutritious diets. Similar to households in the West Bank, head of household education level is associated with a positive change in FCS, reinforcing the importance of education in preventing food insecurity even when facing the challenges that are experienced by households residing in conflict-affected and fragile settings.

In both West Bank and Gaza, households reporting only “minor” impact from mobility constraints experienced a significant decline in FCS relative to those with no impact, whereas the “very much” group displayed a non-significant increase—an unexpected direction likely driven by limited power in this small subgroup. These findings hint at a non-linear relationship between mobility barriers and dietary adequacy, warranting confirmation in larger, more granular studies.

Given the increasing number of unacceptable FCS over the survey years, it is imperative for interventions to be targeted and tailored to the Palestinian population in the oPt. Strategies that enhance mobility, improve economic opportunities, provide educational resources, and promote ceasefire could be vital in improving food security for Palestinians. Additionally, continued support for refugees and effective distribution of humanitarian aid are essential to address the immediate needs while working towards long-term solutions to enhance food security in the region; this aspect is in line with findings from previous work that suggests aid may mitigate food insecurity in the region [12]. One of the potential explanations for the higher-than-expected FCS is that over the last decade, organizations such as the World Food Programme, United Nations Children Fund (UNICEF), and the United Nations Relief and Works Agency for Palestine Refugees in the Near East (UNRWA) have provided food assistance or cash assistance to the most vulnerable. There is criticism that humanitarian aid hinders Palestinian efforts to localize food production—so solutions that not only provide aid and support but also allow the involvement of local communities are needed and should be prioritized to ensure sustainability of any improvements to food security and dietary diversity [21].

### Limitations

The use of survey data may introduce biases inherent to self-reported surveys, and the inability to account for individual-level dietary intake data limits the granularity of the analysis. The observational nature of the serial cross-sectional study design prohibits causality, since data was collected at different points in time without tracking the same households. This design is susceptible to cohort effects, where changes may be due to the sampled population than true temporal trends. The potential for measurement error due to data collection changes between survey years. Despite these limitations, the data provide a robust, representative sample and a broad scope of variables for secondary analysis. Serial cross-sectional studies are a useful tool for identifying associations and trends within populations, though they should be supplemented with longitudinal data for more robust conclusions; future research could employ longitudinal cohort studies analyzed alongside ACLED data to elucidate causal relationships between socio-economic factors, violence caused by conflict, and dietary diversity in occupied Palestine.

## Conclusions

The results of this study provide valuable insights into the factors affecting FCS in occupied Palestinian territory, highlighting the long-term trend and significant association of household location and head of household demographics in a protracted conflict setting. The data and analyses underscored the persistent and growing challenges faced by different demographic groups in the oPt. These findings provide a clear call to action for both local authorities and international stakeholders to prioritize humanitarian aid and reinforce food security as well as advocate for cease fire, demilitarization, and peace.

## Data Availability

Due to data ownership and confidentiality agreements the survey and data are not publicly available. As mentioned in the “Ethics approval and consent to participate” section, the data used in this study were obtained from a survey designed by the Palestinian Central Bureau of Statistics for census purposes that do not require formal informed consent to participate and was exempt from the UCSF IRB approval (24-40704). However, summary statistics and analysis methods are available upon reasonable request.

## Abbreviations

FCS: Food consumption score
oPT: Occupied Palestinian territory
Gaza: Gaza strip
WFP: World Food Programme
ACLED: Armed conflict location and event data
CI: Confidence interval
UNICEF: United Nations Children Fund
UNRWA: United Nations Relief and Works Agency for Palestine Refugees in the Near East
UCSF: University of California, San Francisco
IRB: Institutional Review Board
